# Efficacy and safety of target combined chemotherapy in advanced gastric cancer: a meta-analysis and system review

**DOI:** 10.1186/s12885-016-2772-5

**Published:** 2016-09-15

**Authors:** Kun Zou, Shuailong Yang, Liang Zheng, Chaogang Yang, Bin Xiong

**Affiliations:** Department of Oncology, Zhongnan Hospital of Wuhan University, Hubei Key of Laboratory of Tumor Biological Behaviors & Hubei Cancer Clinical Study Center, Wuhan, 430071 People’s Republic of China

**Keywords:** Advanced gastric cancer, Target drugs, Chemotherapy, Meta-analysis

## Abstract

**Background:**

The aim of our meta-analysis is to assess the efficacy and safety of the target combined chemotherapy for the patients with unresectable advanced or recurrent gastric cancer.

**Methods:**

In accordance with the standard meta-analysis procedures, the patients included in our study were with unresectable advanced or recurrent gastric cancer and allocated randomly to receive target combined chemotherapy or the traditional chemotherapy. The search was applied to PubMed, EMBASE, Science Citation Index Expanded, Cocran’s library (from inception to February 2016). All analyses were performed by STATA 12.0, with the odds ratio, hazard ratio, and 95 % confidence interval as the effect measures.

**Results:**

Fourteen studies were included in this meta-analysis. A total of 5067 patients with advanced gastric cancer were divided into two arms: traditional chemotherapy arm and target combined chemotherapy arm. A significant improvement for overall survival (hazard ratio was 0.89, 95 % confidence interval: 0.83–0.95) and overall response rate (odds ratio was 1.44, 95 % confidence interval: 1.15–1.81) was observed, but no significant difference was found for progression-free survival (hazard ratio was 0.89, 95 % confidence interval: 0.77–1.00) in the target combined chemotherapy arm. In subgroup analysis, increasing benefits regarding overall survival and progression-free survival were found in anti epidermal growth factor receptor target drugs for selected patients subgroup and anti vascular endothelial growth factor receptor target drugs for unselected patients subgroup, but not in anti epidermal growth factor receptor target drugs for unselected patients subgroup. Besides, some adverse events were increased in the target combined chemotherapy arm.

**Conclusions:**

The target combined chemotherapy represented a better overall survival benefit and treatment efficiency and higher incidence of some grade 3–4 adverse events than the traditional chemotherapy for patients with unresectable advanced or recurrence gastric cancer. The anti vascular endothelial growth factor receptor drugs can improve the efficacy in the whole patients with unresectable advanced or recurrence gastric cancer and the anti epidermal growth factor receptor target drugs can only improve the efficacy in the epidermal growth factor receptor positive patients.

## Background

Gastric cancer is the fifth most common malignant neoplasm and the third leading cause of death related to cancer [1]. Surgery remains the only potentially curative treatment in early stage adenocarcinoma, and multidisciplinary treatment has improved the prognosis in radically resectable disease [2]. However, most of patients show an advanced disease or distant metastasis at diagnosis and lose the opportunity of surgical treatment. At the same time, most of the patients relapsed after a prior curative surgical approach. The prognosis is very poor in these patients and chemotherapy represents the reference treatment determining a significantly higher survival benefit compared with the best supportive care alone [3]. However, despite the use of the latest chemotherapy regimen, the median survival time of patients with advanced gastric cancer was only about 10 months [4]. Therefore, it is important to change the existing strategies and find more effective combination chemotherapy for the advanced gastric cancer.

In order to improve the effectiveness of treatment for patients with advanced gastric cancer, scores of efforts has been made. In recent years, with the tumor molecular biology, genetic mechanism and epigenetic association studies gradually expanded, more and more target drugs have been used on the treatment of advanced gastric cancer. As the important signaling pathway of tumor growth and progression, epidermal growth factor receptor (EGFR) and vascular endothelial growth factor receptor (VEGFR) are closely related to tumor invasiveness, studies have confirmed that the expression of EGFR and VEGFR was related to the poor prognosis of gastric cancer [5, 6]. Therefore, anti-EGFR and anti-VEGFR target drugs for the treatment of advanced gastric cancer may be of great significance. Trastuzumab was the first molecule-targeted agent approved for the treatment of gastric cancer after the randomized, prospective, multicenter, phase 3 (ToGA) study. The ToGA study demonstrated that anti-EGFR target drugs (trastuzumab) combined chemotherapy achieved a significant survival benefit compared with the traditional chemotherapy [7]. Subsequently, more anti-EGFR and anti-VEGFR target drugs were used for the treatment of advanced gastric cancer. According to the good survival benefit of the TOGA [7] and RAINBOW [8] clinical trials, the NCCN (National Comprehensive Cancer Network) guidelines for gastric cancer in year 2015 recommends truastuzumab can be used as first-line chemotherapy for patients with human epidermal growth factor receptor-2 positive advanced gastric cancer, and points out that ramucirumab can be used for advanced gastric cancer patients after the failure of first-line chemotherapy. On the other hand, some other clinical trials [9–13] showed that target combined chemotherapy did not achieve any significant survival benefit compared to the traditional. Currently, the efficacy and safety of target drug for the treatment of advanced gastric cancer remains controversial.

At present, along with the development of evidence-based medicine, world medical researchers generally accept the large scale, multicenter randomized clinical trials (RCTs) and the combination of meta-analysis as the best evidence. Therefore, we made a summary of the studies about the treatment of the target drugs for advanced gastric cancer, and carried out a meta-analysis in order to provide reference for clinical chemotherapy of advanced gastric cancer.

## Methods

### Search strategy

According to the relevant requirements of Cochrane collaboration network and the proposed search strategy, two authors used a broad search strategy independently with key words “gastric cancer”, “target” and “chemotherapy” in PubMed, EMBASE, Science Citation Index Expanded, Cocran’s library (from inception to February 2016). An additional search through Google Scholar and the clinical trials registration website was conducted to obtain information about the registered trails. Discrepancies were resolved by the third author. In order to ensure the integrity of the retrieval, we also conducted a manual search.

### Inclusion and exclusion criteria

Patients with gastric cancer involved in the studies were: (1) Confirmed by endoscopic biopsy and postoperative pathological examination; (2) No other primary tumor treatment history; (3) No significant difference of basic information was existed between patients when random assigned; (4) No obvious chemotherapy taboo and liver and kidney dysfunction; (5) Performance status (PS)≦2.

The inclusion criteria were as follows: (1) studies aimed to compare efficacy or safety between target combined chemotherapy and the traditional chemotherapy as the treatment for patients with advanced gastric adenocarcinoma (unresectable, recurrent or metastatic gastric cancer); (2) data for calculating the efficacy or safety of these two therapies were enough. The sufficient data to calculate a hazard ratio (HR) and 95 % confidence interval (95 % CI) for overall survival (OS) and progression-free survival (PFS) and a odds ratio (OR) with 95 % CI for overall response rate (ORR) and adverse events should be available; (3) randomized controlled trials of II phase and III phase; (4) more than 20 patients involved in the trails; (5) articles published in English language; (6) Similar studies or the same research retrieved the recently published article.

The exclusion criteria were: (1) studies with insufficiently data for efficacy and safety including protocols and phase I clinical trials; (2) studies based on overlapping patients; (3) meta-analysis, cohort study, review, single test, case report, conference reports and experiments, report of the expert experience; (4) outcome was unclear or the apparent paradox existed; (5) after contacting the original author or magazine, it still cannot get enough data; (6) target drugs monopoly or different target drugs combined chemotherapy; (7) articles published in non-English language books or papers or the publication of the lack of credibility.

### Data extraction and outcomes

Data retrieved from the publications included: author’s name, year of publication, treatment, number of patients, age, sex, liver function, kidney function, genes that drugs targeted, country (or area) of patients etc. Primary outcomes were overall survival, progression-free survival and overall response rate. Secondary outcomes were adverse events. All data was extracted independently by two investigators, and any discrepancy between the reviewers was resolved by consensus. As all tudies were randomized controlled trials, we summarized the basic information and scored the studies, according to the Cochrane manual scoring standard. This article followed the QUORUM and the Cochrane Collaboration guidelines (http://www.cochrane.de) for reporting meta-analysis (PRISMA statement).

### Statistical analysis

All data in our meta-analysis were analysed by using the STATA 12.0 package (StataCorp, College Station, TX, USA). HR with 95 % CI was used for PFS and OS as demonstrated by Parmar MK et al. and HR > 1 reflects more deaths or progression in the target combined chemotherapy arm [14]. For binary data, including ORR and adverse events, OR with 95 % CI was used and a benefit outcome for chemotherapy response or an unfavorable outcome for adverse events was found in the target combined chemotherapy arm when OR > 1. Heterogeneity was assessed by *I*^2^ inconsistency test and *χ*^2^-based Cocran’s Q statistic test in which *I*^2^ > 50 %, or *P* < 0.05 indicated significant heterogeneity. All studies included in this paper are randomized controlled trials, so when *I*^2^ < 50 %, the fixed effect model was used, or the random effects model conversely. The subgroup analysis was performed when necessary (such as large heterogeneity, *I*^2^ > 50 %). Publication bias was detected by Begg’s test and Egger’s test. *P* < 0.05 was considered significant.

## Results

### Eligible studies

**Fig. 1:**
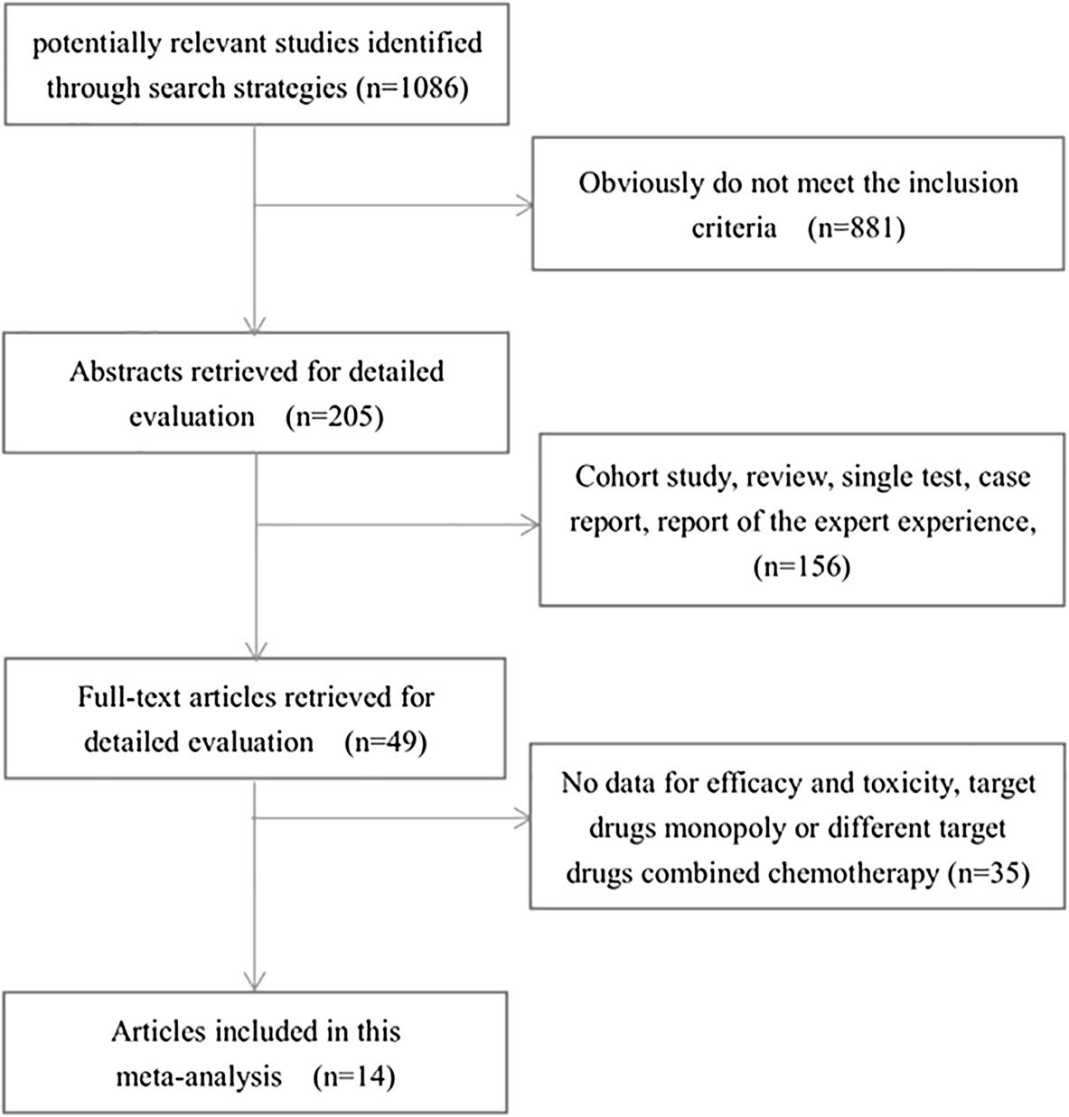
Meta-analysis profile summarizing trail flow

According to the retrieval method mentioned above, 1086 potentially relevant studies were assessed. Detailed steps of search are shown in Fig. 1. After the selection procedure, fourteen studies were included [7–10, 12, 13, 15–22], with a total of 5067 patients with advanced gastric cancer (2539 patients in the target combined chemotherapy arm and 2528 patients in the traditional chemotherapy arm). There was no significant difference in the baselines (such as age, gender, tumor location, PS score, country, etc.). The basic characteristics of these studies were showed in Table 1. For the retrieved fourteen studies, ten were for the first-line chemotherapy and four were for the second-line chemotherapy. In terms of the patient’s status, ten studies were designed for the unselected (whole) advanced gastric cancer patients and four studies were for the selected (EGFR-positive). And we also noted that patients in seven studies came from Asia, two from Europe, four from worldwide, while one’s information is unknown [15]. As to the difference of the target drugs, eight studies used the anti-EGFR target drugs and the six remaining studies used the anti-VEGFR target drugs. All of the included studies were of high quality (Table 2).

**Table 1:** Basic characteristics of the studies included in this meta-analysis

Study	Regimen	PN	Area	TP	TG	Patient
Bang Y. J 2010 [7]	Trastuzumab + fluoropyrimidine + cisplatin	294	worldwide	FL	EGFR	Selected
	fluoropyrimidine-cisplatin	290				
Wilke H 2014 [8]	Ramucirumab + paclitaxel	330	Asia	SL	VEGFR	Unselected
	Placebo + paclitaxel	335				
Shen L 2015 [9]	Bevacizumab + Cisplatin + Capecitabine	100	Asia	FL	VEGFR	Unselected
	Placebo + Cisplatin + Capecitabine	102				
Lordick F 2013 [10]	Cetuximab + Capecitabine + cisplatin	455	worldwide	FL	EGFR	Unselected
	Capecitabine + cisplatin	449				
Waddell T 2013 [12]	Panitumumab + mEOC	278	Europe	FL	EGFR	Unselected
	Epirubicin + Capecitabine + oxaliplatin	275				
Rao S 2010 [13]	matuzumab + Epirubicin + Capecitabine + cisplatin	35	Europe	FL	EGFR	Selected
	Epirubicin + Capecitabine + cisplatin	36				
NCT01246960 [15]	Ramucirumab + Oxaliplatin + Leucovorin + 5-Fu	84	N	FL	VEGFR	Unselected
	Placebo + Oxaliplatin + Leucovorin + 5-Fu	84				
Ohtsu A 2011 [16]	Bevacizumab + fluoropyrimidine + cisplatin	387	worldwide	FL	VEGFR	Unselected
	Placebo + fluoropyrimidine-cisplatin	387				
Du F 2015 [17]	Nimotuzumab + S-1 + Cisplatin	31	Asia	FL	EGFR	Unselected
	S-1 + Cisplatin	31				
Koizumi W 2013 [18]	TSU-68 + S-1 + cisplatin	45	Asia	FL	VEGFR	Unselected
	S-1 + cisplatin	46				
Satoh T 2015 [19]	Nimotuzumab + irinotecan	40	Asia	SL	EGFR	Unselected
	Irinotecan	42				
Satoh T 2014 [20]	lapatinib + paclitaxel	132	Asia	SL	EGFR	Selected
	Paclitaxe	129				
Yi J. H 2012 [21]	Sunitinib + docetaxel	56	Asia	SL	VEGFR	Unselected
	Docetaxel	49				
Hecht J. R 2016 [22]	Lapatinib + Capecitabine + Oxaliplatin	272	worldwide	FL	EGFR	Selected
	Placebo + Capecitabine + Oxaliplatin	273				

*PN* patient number, *TP* treatment plan, *TG* the targeted gene, *FL* first-line, *SL* second-line, *Selected* the EGFR-positive, *unselected* the whole patients, *N* unknown

**Table 2:** Quality assessment of RCTs by Cochrane manual scoring standard

Studies	Design	RM	Blinding	Follow-up	DT	Quality
Bang Y. J 2010 [7]	RCT	IVR	Single	Yes	Yes	A
Wilke H 2014 [8]	RCT	IVR	Double	Yes	Yes	A
Shen L 2015 [9]	RCT	IVR	Double	Yes	Yes	A
Lordick F 2013 [10]	RCT	IVR	Single	Yes	Yes	A
Waddell T 2013 [12]	RCT	IVR	Single	Yes	Yes	A
Rao S 2010 [13]	RCT	IVR	Single	Yes	Yes	A
NCT01246960 [15]	RCT	N	Double	Yes	Yes	B
Ohtsu A 2011 [16]	RCT	IVR	Double	Yes	Yes	A
Du F 2015 [17]	RCT	IVR	Single	Yes	Yes	A
Koizumi W 2013 [18]	RCT	IVR	Single	Yes	Yes	A
Satoh T 2015 [19]	RCT	RPD	Single	Yes	Yes	A
Satoh T 2014 [20]	RCT	IVR	Single	Yes	Yes	A
Yi J. H 2012 [21]	RCT	IVR	Single	Yes	Yes	A
Hecht J. R 2016 [22]	RCT	IVR	Double	Yes	Yes	A

*IVR* interactive voice recognition system, *RPD* random permuted blocks, *RM* randomization method, *DST* description of test methods

### Progression-free survival (PFS)

**Fig. 2:**
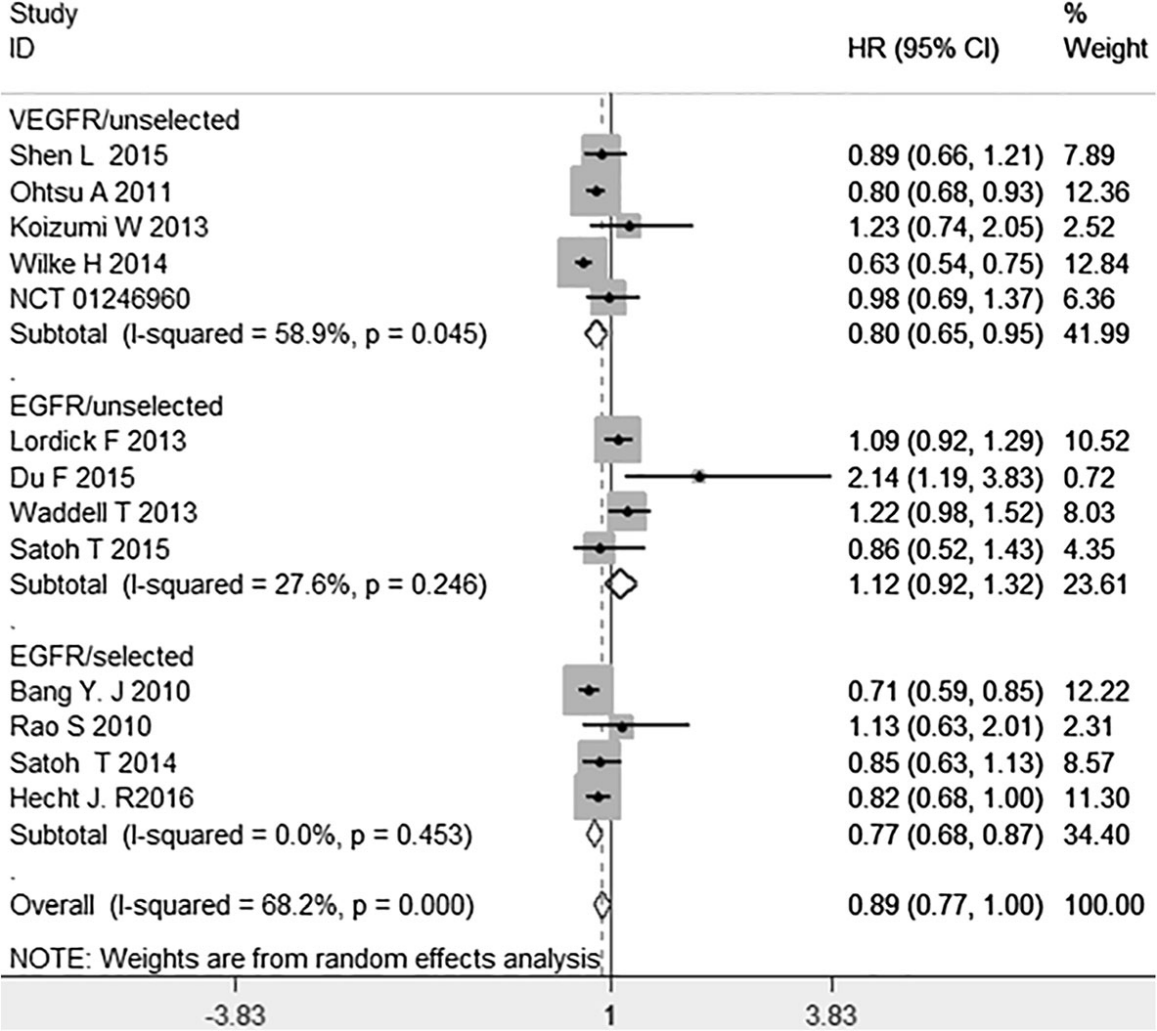
Hazard ratio (HR) for progression-free survival (OS) of the included trials

Data on progression-free survival was available in thirteen studies (4962 patients), there was an significant heterogeneity for PFS between these studies when pooling the HR. Then we pooled the HR in random-model and found no significant improvement for PFS (HR = 0.89, 95 % CI: 0.77–1.00, *P* = 0.055; *I*^2^ = 68.2 %). Depending on the difference of the target drugs and patient’s status that whether they were selected or not, when entering the studies, the studies were divided to three subgroups. The pooled HR was 0.80 (95 % CI: 0.65–0.95, *P* = 0.009) in anti-VEGFR target drugs for unselected patients subgroup, 1.12 (95 % CI: 0.92–1.32, *P* = 0.228) in anti-EGFR target drugs for unselected patients subgroup, 0.77 (95 % CI: 0.68–0.87, *P* < 0.001) in anti-EGFR target drugs for selected (EGFR-positive) patients subgroup (Fig. 2). A significant improvement for PFS was found in anti-VEGFR target drugs for unselected and anti-EGFR target drugs for selected patient subgroup, not in anti-EGFR target drugs for unselected patient subgroup.

### Overall survival (OS)

**Fig. 3:**
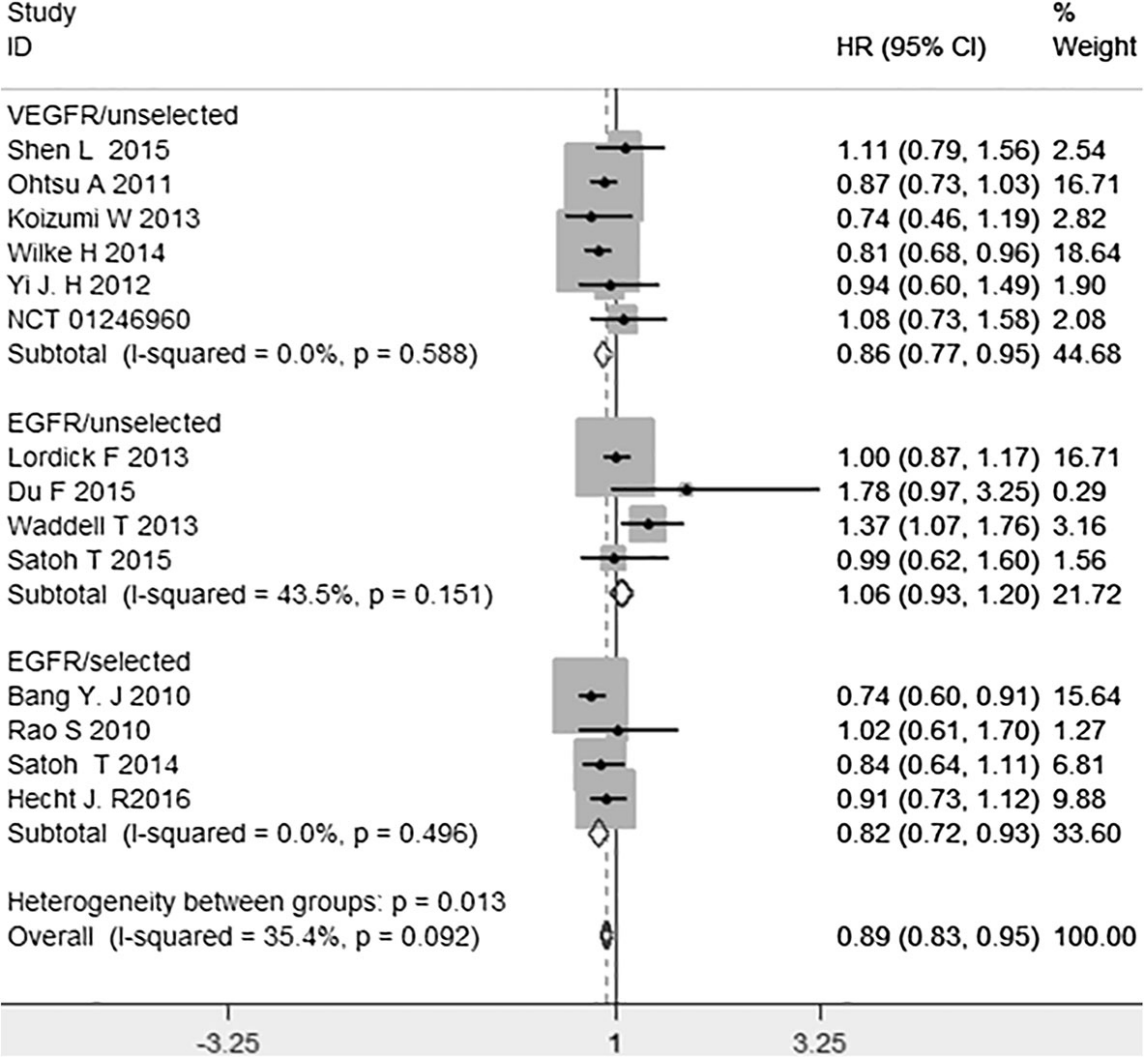
Hazard ratio (HR) for overall survival (OS) of the included trials

All the fourteen studies demonstrated OS. The *I*^2^ value of heterogeneity test was 35.4 % and a fixed-effect model was used. The pooled HR was 0.89 (95 % CI: 0.83–0.95, *P* = 0.001). The difference had statistically significance. There was an significant benefit and a risk reduction of death by 11 % (HR = 0.89) in target combined chemotherapy arm, compared to the traditional chemotherapy arm. As to subgroup analysis, a significant improvement for OS was found in anti-VEGFR target drugs for unselected patients subgroup (HR = 0.86, 95 % CI: 0.77–0.95, *P* = 0.003), and anti-EGFR target drugs for selected patients subgroup (HR = 0.82, 95 % CI: 0.72–0.93, *P* = 0.001). But there was no significant improvement for OS in anti-EGFR target drugs for unselected patients subgroup (HR = 1.06, 95 % CI: 0.93–1.20, *P* = 0.342) (Fig. 3).

### Overall response rate (ORR)

**Fig. 4:**
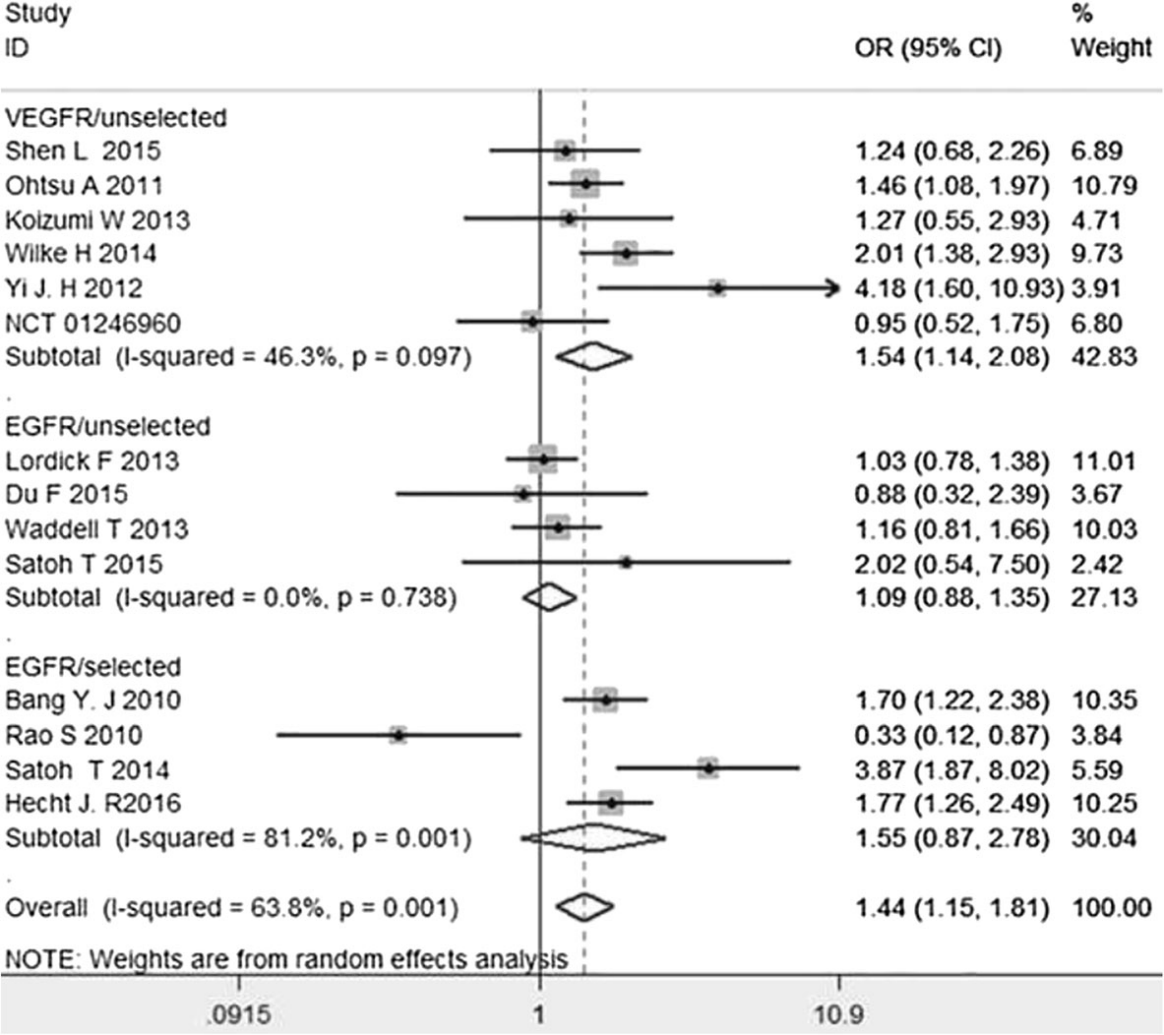
Odds ratio (OR) for overall response rate (ORR) of the included studies. Abbreviations:hazard ratio (HR); 95 % confidence interval (95 % CI); anti epidermal growth factor receptor target drugs for selected patients subgroup (EGFR/selected); anti vascular endothelial growth factor receptor target drugs for unselected patients subgroup (EGFR/unselected); anti epidermal growth factor receptor target drugs for unselected patients subgroup (VEGFR/unselected)

All the fourteen studies demonstrated ORR. The ORR of the target combined chemotherapy arm ranged from 17.5 % to 62.2 %, while the ORR of traditional chemotherapy arm ranged from 8.5 % to 58.3 %. The *I*^2^ value of heterogeneity test was 63.8 % and a random-effect model was used. The meta-analysis shown an significant improvement for ORR in target combined chemotherapy arm (OR = 1.44, 95 % CI: 1.15–1.81, *P* = 0.002). Similarly, a significant improvement for ORR was found in anti-VEGFR target drugs for unselected patients subgroup (OR = 1.54, 95 % CI: 1.14–2.08, *P* = 0.005). But, consistent result was found between two arms in the subgroups of anti-EGFR target drugs for selected patients subgroup (OR = 1.09, 95 % CI: 0.88–1.35, *P* = 0.433) and anti-EGFR target drugs for unselected patients subgroup (OR = 1.55, 95 % CI: 0.87–2.78, *P* = 0.140) (Fig. 4).

### Grade 3 to 4 adverse events

Due to the difference of the adverse events reported in the studies, we summarized the grade 3–4 adverse events that at least reported in five trials and the results were showed in Table 3. For the whole adverse events of 3–4 grade, data was available in six of the trials and the pooled OR was 1.53 (95 % CI: 0.98–2.40, *P* = 0.062). There was no significant difference in the incidence of the whole grade 3–4 adverse events between two arms. Similarly, there was no difference in the grade 3 to 4 hematological adverse events (such as Anemia, Thrombocytopenia, neutropenia, leucopenia). Only constipation was decreased in the target combined chemotherapy arm and diarrhea, fatigue, the skin and subcutaneous tissue disorders (rash, hand-foot syndrome, stomatitis) shown a significant difference between two arms.

**Table 3:** Grade 3 to 4 adverse events of interest

AEs	*I* ^*2*^	OR	95 % CI	*P* value
Anemia	0.0 %	1.02	0.83–1.24	0.883
Thrombocytopenia	39.2 %	1.06	0.74–1.51	0.771
Leucopenia	66.1 %	1.24	0.73–2.10	0.433
Neutropenia	85.8 %	1.33	0.88–2.01	0.179
Febrile neutropenia	67.2 %	1.34	0.68–2.67	0.401
Hypokalemia	38.1 %	1.09	0.81–1.47	0.586
Rash	0.0 %	21.84	6.81–70.02	<0.001*
Hand-foot syndrome	37.6 %	1.75	1.22–2.50	0.002*
Stomatitis	42.8 %	2.08	1.23–3.51	0.006*
Nausea	21.6 %	0.94	0.73–1.21	0.639
Vomit	0.3 %	0.99	0.79–1.25	0.944
Abdominal pain	0.0 %	1.10	0.47–2.57	0.821
Diarrhea	14.1 %	2.40	1.88–3.06	<0.001*
Constipation	0.0 %	0.27	0.08–0.91	0.034*
Decreased appetite	0.0 %	1.13	0.88–1.46	0.342
Fatigue	0.0 %	1.55	1.21–2.00	0.001*
Neuropathy	62.8 %	0.76	0.24–2.41	0.636
Any adverse events	86.8 %	1.53	0.98–2.40	0.062

*P < 0.005, There were statistically significant differences between the two arms

### Publication bias

Publication bias was detected by Begg’s test and Egger’s test. *P* < 0.05 confirmed the existence of publication bias. No publication bias was shown in OS (Begg’s *P* = 0.324, Egger’s *P* = 0.279) and ORR (Begg’s *P* = 0.827, Egger’s *P* = 0.936). PFS showed borderline publication bias by Begg’s test (*P* = 0.044), however, no publication bias by Egger’s test (*P* = 0.108).

## Discussion

Systematic chemotherapy is the basic treatment for patients with advanced gastric cancer, and it can achieve better survival benefits compared with single drug chemotherapy or best supportive care [3]. But, for the patients with advanced or metastatic gastric cancer, the effect of combined chemotherapy is limited. Studies have found that, despite of the development of new chemotherapy regimens, the 5-year survival of patients in this setting was less than 10 %, median survival time was only 10–13 months [23]. In recent years, it is thought that VEGFR and EGFR play a crucial role in the growth of most primary tumors and the subsequent process of metastasis. The expression of VEGFR and EGFR was an independent prognostic indicator of worse outcome in gastric cancer patients [24]. EGFR has been identified widely expressed in a variety of tumors and about 20 % to 30 % of patients with gastric carcinoma overexpress EGFR [6]. Therefore, application of EGFR and VEGFR molecular targeted drugs may provide a new direction for treatment of advanced gastric cancer. A study with truastuzumab combined chemotherapy showed favorable efficacy of a 68 % response rate, 16 months of OS, and 7.8 months of PFS in EGFR-positive advanced gastric cancer [25]. In another clinical trial, ramucirumab (anti-VEGFR target drugs) monopoly chemotherapy resulted significant survival benefit compared with the best support care [26]. Moreover, the expression of related genes may affect the therapeutic effect of target combined chemotherapy for patients with advanced gastric cancer. The EXPAND trail shown that the selected (EGFR-positive) patients has better improvement for ORR than the unselected when treated with target combined chemotherapy [10]. The subgroup analysis of AVAGAST trail shown that target combined chemotherapy has significant benefit for OS in the selected (VEGFR-positive) patients (HR = 0.72; 95 % CI: 0.57 to 0.93) [27] and that was not noted in unselected patients (HR = 0.87; 95 % CI: 0.73 to 1.03) [16].

Our meta-analysis was conducted for the main purpose of assessing the possible benefit in terms of OS, PFS and ORR by adding anti-EGFR or anti-VEGFR target drugs to chemotherapy. Overall, we noted that the target combined chemotherapy arm had significant improvement for OS and ORR. Therefore, we think the target combined chemotherapy has better overall survival benefit and treatment efficiency than the traditional chemotherapy for patients with unresectable advanced or recurrence gastric cancer. Regarding to that the two arms had significant heterogeneity for PFS and ORR (*I*^2^ was 68.2 % and 63.8 % respectively) and there were no significant improvement for PFS (HR = 0.89, 95 % CI: 0.77–1.00, *P* = 0.055) between two arms, we grouped the studies into three subgroups according to the difference of the drugs and patient’s status. The PFS and OS were significantly improved in anti-VEGFR target drugs for unselected patient subgroup and anti-EGFR target drugs for selected (EGFR-positive) patient subgroup, but no significant improvement was found in anti-EGFR target drugs for unselected patient subgroup. This difference may due to the different mechanism of anti-EGFR and anti-VEGFR target drugs and the limited clinical trials in subgroups. It indicated that anti-VEGFR target drugs can prolong the life time of patients with gastric cancer, but anti-EGFR drugs can only prolong the life time of the EGFR-positive patients with advanced gastric cancer. For ORR, The result showed significantly improved in anti-VEGFR target drugs for unselected patient subgroup, but no difference in anti-EGFR target drugs for selected and anti-EGFR target drugs for unselected patient subgroup. The difference may be explained by the limited clinical trials (only four trails were involved in this subgroup), and the result in anti-EGFR target drugs for selected patients subgroup is greatly influenced by the data by Rao et al. (OR = 0.33, 95 % CI: 0.12–0.87) [13]. When we omitted the data by Rao et al., we noted an significantly improvement for ORR in anti-EGFR target drugs for selected patient subgroup (OR = 1.46, 95 % CI: 1.27–1.67). That the different result was greatly influenced by the data by Rao et al. was also found in another meta-analysis [28].

For grade 3–4 adverse events, we noted that the target combined chemotherapy arm had an obviously increased incidence of the skin and subcutaneous tissue disorders and diarrhea. This was also found in another meta-analysis [28] and some other clinic trails [10, 29, 30]. The skin and subcutaneous tissue disorders may be the special adverse events for the target drugs and we may need to prevent its occurrence when using target drugs. There was no significant difference in the incidence of grade 3–4 blood and lymphatic system adverse events of the two groups, and it means the safety of the blood system of target combined chemotherapy arm is equivalent to chemotherapy alone arm. For all of the outcomes, the large-scale multicenter randomized clinical trials are still needed.

Our meta-analysis has some limitations. Firstly, the meta-analysis used the pooled data which came from published papers, not original data. Secondly, there were several target drugs in this meta-analysis, and the basic chemotherapy, treatment strategy and duration were also different. These all contribute to the high heterogeneity of this meta-analysis. Thirdly, as demonstrated in some trails [19, 22, 31], the improvement of patients from different regions is distinct. The sub-analysis in RAINBOW and ToGA trails demonstrated different improvement in OS between Asian and the whole patients, and the HR for Asian patients was 0.99 (95 % CI: 0.73–1.34) [31] and 0.82 (95 % CI,: 0.61 to 1.11) [11] respectively, however, there were significant improvement in OS in the whole patients. In this meta-analysis, we do not consider the effect of regional differences on the results. Moreover, as shown in AVAGAST trail, the OS benefit was achieved in the selected but not the whole patients, it is possible that the significant improved efficacy of anti-VEGFR target drugs may be justly achieved by refining the selection of the patient population [16]. Little studies were designed for the VEGFR-positive patients with advanced gastric cancer. At the same time, there is little literature to provide quality of life and the cost of treatment between the two arms, so this meta-analysis did not carry out the relative conclusion.

In conclusion, this meta-analysis indicated the target combined chemotherapy was associated with significant improvement for OS and ORR compared with traditional chemotherapy. The addition of anti-VEGFR target drugs to chemotherapy for gastric cancer significantly improved outcome of OS, PFS, ORR, while anti-EGFR target drugs only led to improved outcome for OS and PFS in EGFR-positive gastric cancer. Some adverse events were increased in target combined chemotherapy arm. We recommended anti-VEGFR target drugs can be used for the whole patients with gastric cancer, and anti-EGFR drugs should be selected to EGFR-positive patients. Meanwhile, more high-quality randomized controlled trials are needed to provide more information. And whether the efficacy of anti-VEGFR target drugs is justly achieved by refining the selection of the patient population or not need further research.

## Conclusions

Generally speaking, the target combined chemotherapy represented a better overall survival benefit and treatment efficiency and higher incidence of some grade 3–4 adverse events than the traditional chemotherapy for patients with unresectable advanced or recurrence gastric cancer. The anti-VEGFR drugs can improve the efficacy for the whole patients with unresectable advanced or recurrence gastric cancer and the anti-EGFR drugs can only improve the efficacy for the EGFR-positive patients.

## Abbreviations

95 % CI: 95 % confidence interval
EGFR: Epidermal growth factor receptor
HR: Hazard ratio
OR: Odds ratio
ORR: Overall response rate
OS: Overall survival
PFS: Progression-free survival
RCTs: Randomized clinical trials
VEGFR: Vascular endothelial growth factor
